# Staying “One Step Ahead of a Racist”: Expanding Understandings of the Experiences of the Covid-19 Pandemic Among People From Minoritized Ethnic Groups Living in Britain

**DOI:** 10.3389/fsoc.2021.730313

**Published:** 2021-11-01

**Authors:** Saffron Karlsen, Rosie Nelson

**Affiliations:** School of Sociology, Politics, and International Students, University of Bristol, Bristol, United Kingdom

**Keywords:** Covid-19, coronavirus, ethnicity, health, inequality, race, racism

## Abstract

Covid-19 has had a disproportionate impact on those in minoritized ethnic groups. Considerable attention has been given to evidence of ethnic inequalities in rates of infection, hospitalisation, and death. But other ways in which the pandemic experience has been affected by ethnicity have received less consideration. This paper explores the lived experiences of people in different minoritized ethnic groups living in South West England, during the United Kingdom’s first pandemic lockdown, using qualitative data collected from interviews and comments provided on a survey. Perceived positive opportunities for growth were offset by anxiety and stress, which were themselves compounded by an awareness of the additional risks they experienced as members of racialised groups, and a sense that this was being ignored—or intentionally exacerbated—by the British authorities. Frustration with an incompetent and corrupt national Government was intensified by concerns regarding their racist motives. Racism in wider society undermined confidence in key public institutions, such as the NHS and the police, while also producing barriers to informal local-community pandemic responses. Only through recognition of the particular ways in which the pandemic affected those in minoritized ethnic groups, including the multiple and compounding effects of current and historical racism, will it be possible to identify avenues for transformative systemic policy change and opportunities to rebuild trust and a better post-pandemic society for all.

## Introduction

The evidence regarding the disproportionate impact of the Covid-19 pandemic on those in minoritized ethnic groups living in Britain and elsewhere is irrefutable (Aldridge et al., 2020; Haque et al., 2020; Larsen et al., 2021; PHEd 2020a; 2020b; Platt and Warwick 2020a; Sze et al., 2020; WEC, 2020). In particular, studies have shown those with Bangladeshi, Black African, Black Caribbean, Indian and Pakistani ethnicities to experience higher rates of infection, hospitalisation and death compared with the white British population. But it is clear that the impact of the pandemic on society has not only been in relation to increased illness and death. Increasingly, empirical evidence has exposed other ways in which the pandemic, and the measures introduced to mitigate its effects, have disadvantaged those in minoritized ethnic groups, often by exacerbating pre-existing inequalities (Karlsen et al., 2020; Li and Heath, 2018, Longhi 2018). For example, people in ethnic minority groups already struggling to make ends meet found themselves in occupations more vulnerable to the economic consequences of social distancing measures or less open to working at home or furlough (BSWN, 2020, Platt and Warwick, 2020a,b). The “digital divide” (and implications of overcrowded accommodation) became even more significant in a world where work and study were conducted almost entirely online (Baker et al., 2020). These negative experiences will, in turn, exacerbate persistent ethnic inequalities in health (Bambra et al., 2020; John et al., 2021; Maddock et al., 2021). For example, in the United Kingdom, people in Black, Asian (and “other white”) groups reported poorer mental health and an increased sense of worry following the first pandemic lockdown^1^ (Barnes and Hamilton, 2020). Research from the US has also indicated higher risk of food scarcity as a consequence of the pandemic among marginalised ethnic groups which is likely to be replicated in the United Kingdom (Siddiqi et al., 2021).

Media and political debate regarding these ethnic inequalities often focused only on the immediate health consequences of the pandemic itself. Moreover, the perceived causes of these inequalities tended to prioritise explanations which focused on genetic or biological issues or the choices and behaviours characteristic of particular ethnic groups. Higher rates of Covid-19 infection were presented as being caused by cultural preferences for multigenerational households, which led to overcrowding (CRED, 2021). Higher rates of death were linked to co-morbidities like diabetes and heart disease, which were perceived to be produced by biological and/or behavioural issues. Not surprisingly, Duffy et al. (2021) found that a significant minority of the British public blamed people in minoritized ethnic groups for their own disadvantage. In the US too, “insidious and potentially racist allusions … emerge[d during discussions of the pandemic,] appearing to blame African Americans as somehow responsible for the relatively large number of cases and deaths from Covid-19 in the United States, stoking age-old tropes, and attributing morbidity and mortality to the behaviours and predispositions of BAME groups” (Bentley, 2020).

Yet consistently, the empirical evidence regarding the causes of these and other ethnic health inequalities shows that these are influenced far more greatly by societal/structural issues, than biological or cultural ones (Karlsen et al., 2019; Baumer et al., 2020; Otu et al., 2020; WEC, 2020; Simms, 2021). Endemic processes of direct and indirect racist societal exclusion operate across lives, and over generations, to limit people’s access to good-quality housing, education, employment and healthcare, each of which operate to produce health and economic disadvantage independently and are also mutually reinforcing (Brynin and Guveli, 2012; Darlington-Pollock and Norman, 2017; Rafferty, 2021; Zwysen et al., 2021). The low incomes, poor living conditions, poor health and other negative experiences of parents also impact on the health and economic outcomes of their offspring, who carry this disadvantage into their own child- and adulthoods, to be further exacerbated by their own experiences of exclusion. While we can identify a role for health conditions such as diabetes and heart disease in Covid-19 outcomes, we cannot divorce these from the impacts of lives lived on low incomes in access to healthy food or the higher levels of stress these circumstances induce. Overcrowded accommodation is an economic problem rather than a choice, and is often accompanied by issues of housing quality which will encourage respiratory and other health conditions (Darlington-Pollock and Norman, 2017). As such, these processes of exclusion concentrate those in minoritized ethnic groups in living and working conditions which expose them to greater risks of Covid-19 infection, and reduce their resilience to its more serious consequences when it occurs (Barnes and Hamilton, 2020; Brynin and Guveli, 2012; BSWN, 2020; Li and Heath, 2018; Longhi, 2020; Mamluk and Jones 2020; Platt and Warwick 2020b; Rafferty, 2021; Simms, 2021).

The United Kingdom Government continues to refute the existence of racism and its role in the generation of inequalities, including those recognised during the Covid-19 pandemic (Bamrah et al., 2021; CRED, 2021). Yet racism is a socially embedded phenomenon which plays a central role in the generation and perpetuation of these ethnic inequalities, directly and indirectly contributing to the limited life chances and premature deaths of those in minoritized ethnic groups (Nazroo and Becares, 2020; b; Gee et al., 2012; Karlsen et al., 2019; Karlsen and Nazroo 2002; Kreiger 2014; Lewis et al., 2015; Paradies et al., 2015; Priest et al., 2013; Williams 2018; Williams et al., 2019). We must recognize not only that such racisms produce the economic inequalities which explain ethnic inequalities in Covid-19 infections and their outcomes but also that interpersonal and societal racism may be exacerbated in times of social stress, with significant consequences to those it victimizes.

The more direct ways racism has affected the experience of the pandemic among racialized people living in the United Kingdom remains underexamined. There is emerging evidence, particularly from the United States, regarding the ways in which the branding of Covid-19 as a “Chinese virus” has increased exposure to inter-personal violence among those considered (East) “Asian” (Coates, 2020; Darling-Hammond et al., 2020; Dhanani and Franz, 2020), and of the negative health consequences of this on both adults and children (Cheah et al., 2020). But Black and other Asian Americans also experienced greater interpersonal racist violence during the pandemic (Ruiz et al., 2020). In the United Kingdom, racially-motivated hate crimes reported to the police rose by 12% in the year to March 2021, constituting three-quarters of the 124,091 hate crimes reported during that period (Home Office 2021). We must better understand the impact that this experience has had on its victims.

We must also recognize that racism manifests in various forms, and is not only experienced as explicitly racist verbal or physical violence or dehumanizing treatment such as that which led to the death of George Floyd from police brutality in Minneapolis, US on May 25, 2020, producing a global response so central to the pandemic experience of many, including those in this study. It is also in the more subtle everyday racisms (Essed 1992), or daily hassles, which are more difficult to measure but remain a constant feature of the lives of many people in minoritized ethnic groups (Williams et al., 2003; Karlsen and Nazroo, 2017). Indeed, racism, unlike other criminal acts, need not be experienced personally for it to produce a sense of threat. As such, racist violence should not be considered simply an attack on an individual person, but an attack on a member of a category or group, “an attack on the community as a whole” (Virdee 1995, *p*. 284). George Floyd’s murder, and knowledge of other racist attacks or racially-motivated social exclusions, can serve to increase a sense of personal threat amongst those in racialised groups. Anticipating experiences of prejudice produces higher levels of stress (including an identifiable cardiovascular response) even where this expectation is not realised in an experience of violence (Sawyer et al., 2012). As such, simply living with the fear of being a victim of racism has found to be significantly associated with poorer health experience (Karlsen and Nazroo, 2004).

Research consistently shows the ways in which experiences of vicarious/indirect racism, and “vigilant coping”–monitoring or modifying your behaviour to protect yourself from anticipated discrimination–can directly affect mental wellbeing, with increased depression, anxiety, sleep deprivation and symptoms of post-traumatic stress disorder (Hicken et al., 2013; Himmelstein et al., 2015; Tynes et al., 2019; Chae et al., 2021). Identified physical responses to indirect racism and vigilant coping include elevated cortisol (Huynh et al., 2017) as well as higher rates of obesity (Hicken et al., 2018) and cardiovascular disease (Clark et al., 2006). The stress of vicarious racism has also been recognised in the rise in adverse health outcomes—such as elevated cortisol, or pre-term and low birthweight birth among those in racialised groups—following events which reinforce a sense of endemic racism in a society (Smart Richman and Jonassaint, 2008)–including the anti-immigration raids in Iowa, US in 2008 (Novak et al., 2017) and the presidential election of Donald Trump in 2016 (Gemmill et al., 2019).

Finally, we must recognize that racism is more than a sum of its parts. Single incidents of racism, while influential in themselves, also evoke painful memories of past racist experiences and communal histories of prejudice, which exacerbate their impact. Evidence from the Pew Research Center that, during the pandemic, Black and Asian Americans were more likely to worry that people will be suspicious of them while wearing a protective facemask in stores or other businesses (Ruiz et al., 2020) suggests that persistent racist tropes of higher criminality among certain minoritized ethnic groups continue to leach into people’s daily lives. Racism identified in one domain can also raise concerns about a risk of exposure in others, particularly for incidents perpetuated by people in positions of societal responsibility. Emerging evidence from the United Kingdom indicates that people in minoritized ethnic groups were exposed to more aggressive policing during the lockdown period (Busby and Gidda, 2020; Harris et al., 2021a). Living in areas with very high levels of police brutality have been found to be significantly and directly associated with poorer mental health and higher blood pressure and obesity, the latter both physical manifestations of extreme stress (Bor et al., 2018; Sewell et al., 2021). But Harris et al. (2021b) argue that “the new police powers [introduced during the pandemic were] adding to and exacerbating pre-existing forms of racist policing”. As such, these recent experiences may also reinforce a preexisting sense of distrust in the police both among the direct victims and others in racialized groups. As a service operating on behalf of, and representing, wider structures of power in society, these experiences will also add to people’s concerns regarding their risks of experiencing unfair treatment in their interactions with other statutory services as well as the extent to which they can expect protection from harm from the Government, in relation to their pandemic vulnerabilities or more generally.

This paper aims to help develop a clearer picture of the experiences of the first pandemic lockdown, using interviews conducted with, and written comments provided by, individuals who considered themselves part of a minoritized ethnic group and living in the South West region of England. While we find evidence of some positive lockdown experiences, stories are often haunted by an awareness of the existence and implications of widespread racially-motivated prejudice and ill-treatment in British society. Some participants describe personal experiences of negative treatment, but a sense of risk of exposure to racist violence also had a significant impact on people’s lives. Pervasive among this sample are negative attitudes towards the pandemic responses of the British Government, and the sense that these were motivated by racism in the highest offices. Rebuilding British society in its aftermath demands that we recognise and respond to this directly.

## Methods

This paper reports qualitative findings from a project exploring the pandemic experiences of people in minoritized ethnic groups living in the South West of England, conducted between June and September 2020. The project was developed in partnership with Black South West Network (BSWN), a Bristol-based Black-led racial justice charity organisation. The study comprised an online survey, distributed via the mailing lists of BSWN and other organisations based in the South West with an ethnically diverse membership. The survey asked a series of questions about people’s lives before and during the pandemic lockdown. Respondents to this survey were invited to participate in a follow-up semi-structured in-depth interview. These interviews explored how participants had spent their time during lockdown, how they had been feeling, whether they had received any help they needed and their attitudes towards the activities of their local community and national and local government during this period. This paper focuses on findings from the nine people who participated in the interviews, and the 56 people (out of the 108 who responded) who provided comments on an open section of the survey. Neither the survey or interview asked people directly about the impact of their ethnicity or racism on these experiences. As a consequence, the centrality of issues of ethnicity/“race” to these discussions/comments varies between them. However, because these issues were raised spontaneously, this offers a more realistic reflection of the importance/relevance of these issues to participants’ perceptions of these experiences than might be offered by studies which ask about these issues more directly.

Pandemic restrictions prevented in-person interviews. Interviews were therefore conducted remotely—*via* phone, Zoom, or Microsoft Teams depending on participant preference, by a researcher presenting as a feminine white person. It is unclear whether, or how, these factors affected the findings. Interviews were only conducted in English and were between 30 and 60 min duration. Following the interviews, participants were sent a list of resources for further information and support and a £10 voucher. Interviews were audio or video recorded, dependent on platform, and uploaded to a secure server before being transcribed and deleted. Interviews were transcribed with all identifiable details anonymized. Ethical approval for the study was provided by the ethics committee of the School for Sociology, Politics and International Studies at the University of Bristol (Ref: SK050620).

The interview participants variously defined their ethnicity as: Black African, British Indian, South African/Mixed Race, Kashmiri, Black British Caribbean, Black Caribbean British, Black Caribbean, and Black British. All interview participants classified themselves as cis women and the majority were born in the United Kingdom. Two participants were aged in their mid-to late 20s, two were in their late 30s or early 40s, four were in their mid-50s and one was in their early 60s. Some participants lived alone, others with their partner and/or children. Most of the participants considered themselves relatively financially secure, and relatively unaffected by the more severe economic implications of the pandemic exposed in other research (Barnes and Hamilton, 2020). Most of them were able to work from home during the Covid-19 lockdown. The nature of the study methodology meant that they also did not suffer from the most severe aspects of digital exclusion. These factors would suggest that exposure to the more negative aspects of the pandemic may have been limited among this group. However, several participants had been unable to find work or lost business opportunities or investments as a result of the economic slowdown coinciding with the pandemic. As such, this experience of privilege is by no means homogeneous or universal. However, these factors reinforce our awareness that, as with all qualitative research, these study findings are potentially unique to this sample and setting and cannot be generalised to other individuals or groups, in the United Kingdom or elsewhere.

The survey respondents providing comments were somewhat more diverse than those participating in the interview. Of the 56 people providing comments, six classified themselves as cis male. One person classified themselves as “Bangladeshi”, five as “Black African”, 13 as “Black Caribbean”, two as “Black – other”, three as “Chinese”, eight as “Indian”, two as “Mixed – Asian and white”, three as “Mixed – Black African and white“, seven as “Mixed – Black Caribbean and white”, two as “Mixed – other”, two as “other Asian”, two as “Pakistani” and one as “Somali”. The five people who classified themselves as “other”, described their ethnicity as (variously): “Taiwanese”; “British Asian/Indian”, “Filipino”, “Jamaican/Indian” and “3/8 Jamaican, 1/8 Indian and 1/2 English” Their inclusion therefore helps mitigate some of the impact of bias produced by the apparent homogeneity of the interview sample.

A thematic analytical approach was used (Braun and Clarke 2006), which examined separately the themes emerging from the interview transcripts and survey comments. In each case, one author took the lead in analysing a source, whose decisions were ratified by the other. The themes from each data source were then combined for presentation. In the findings, quotes from interview participants can be identified by the prefix “IP” in their participant identifier, while survey respondents can be identified by the prefix “SR”. For each quotation, we also include information on the gender, age and self-identified ethnicity of the contributor. Themes identified relate to 1) the impact of ethnicity on people’s pandemic experience, 2) the impact of a heightened sense of Covid-19 risk, and 3) the causes and consequences of inadequate Government responses to the pandemic.

## Results and Discussion

Many participants described the ways in which the lockdown period had provided opportunities for positive personal growth. However, the period was also associated with experiences of loss, personal difficulty and heightened emotion. For those in minoritized ethnic groups, there were additional issues associated with expectations regarding the maintenance of particular cultural traditions and absence from and fear for family living abroad. People’s concerns were also exacerbated by evidence regarding the greater risk of infection and death among those in minoritized ethnic groups and a perceived lack of ability among participants to protect themselves and their family from this. People also expressed a sense of additional vulnerability to racist violence, which people felt had risen in response to the pandemic and other incidents occurring during this time. Racism from others could act as a barrier to support from their local community, while a fear of exposure to racism could also affect people’s social engagement in a range of ways.

Things were made more difficult by what was ubiquitously considered the national Government’s poor handling of the crisis. The measures introduced to protect people—including those specifically targeted at those in minoritized ethnic groups—were considered ineffective. Participants also described the implications of Government and media discourses which were seen to purposefully misrepresent the evidence regarding the drivers of ethnic inequalities in Covid-19 infections and deaths. Not only did this cause confusion amongst those attempting to determine and respond to their personal Covid-19 risk but was also argued to directly increase their risk of exposure to racist violence. Some participants considered the Government’s dismissive attitude to be the latest manifestation of a longstanding racism among political leaders and other powerful bodies in Britain. This awareness exacerbated concerns regarding how to protect themselves from infection and their chances of receiving care if they became ill. It also reinforced a wider sense of marginalisation in and exclusion from British society.

### The Impact of Ethnicity on the Pandemic Experience

Perhaps as a consequence of being largely unencumbered by the socioeconomic stresses identified as disproportionately affecting those in minoritized ethnic groups (Barnes and Hamilton, 2020; Siddiqi et al., 2021), all interview participants described positively the ways in which pandemic lockdown had offered them opportunities for personal and/or social growth. This uninterrupted time enabled people to develop new or reinvigorate old hobbies and “take stock” of and “re-evaluate” their lives. People valued having capacity to pause, be more “mindful”, “slow [their] pace of life” and learn to appreciate “small achievements”. Lockdown also enabled some people to engage in making special memories with significant others, and build stronger networks in their local community.

But alongside these more positive experiences, participants described various difficulties. Several participants had experienced the death of relatives and friends, from Covid-19 and other causes. Participants described loneliness and the impact of the loss of “control”, freedom, valued social contacts and previously-made plans. Some lived in accommodation which was considered too “confined”. Others, particularly those with young children, experienced difficulties due to a lack of outside space. Many participants described an increase in “anxiety” with the “uncertainty” brought by the pandemic, some even fearing “mental breakdown”. People experienced additional stress—going into “overdrive” to manage the new complexities of carrying out ordinary tasks, like visiting the supermarket. There were also simple activities which no longer seemed possible:

It has been really really hard. […] You’re not feeling the same freedom, you used to go out and chat […] I’m really chatty–I would see people on the street and start chatting to them, but obviously you don’t feel the same way. Even if you wanted [to chat], the other person might be apprehensive if she has [in case I had] Covid (IP04, Female, 42, Kashmiri)

Such findings are consistent with research on the pandemic experiences of the general population (Ettman et al., 2020; Reading Turchioe et al., 2020). However, there were particular ways in which being part of a minoritized ethnic group was considered to have impacted on people’s lockdown experience. For example, some people felt that the difficulties of social distancing were exacerbated by cultural traditions encouraging more frequent social contact:

Back home, you’re used to family and friends coming around all the time, which is really different here (IP04, Female, 42, Kashmiri).

I have an Indian background. We are all about family and keeping in touch. […] Not seeing them every week was difficult (IP02, Female, 28, British Indian).

Cultural expectations regarding the provision of care to the infirm, bereaved and deceased also could add to the mental strain of the pandemic, particularly when these traditions were not recognised or responded to by statutory services or policymakers:

Cultural expectations of many South Asian communities require different types of support during illnesses and deaths. E.g Bangladeshi families don’t cook for 3 days post any death in the family. Family and friends are expected to deliver food to the mourning family during this time. Lockdown during this pandemic has increased the burden of community care on many of us. Without any state support and the disproportionate death rates in our community puts us under unprecedented anxiety and stress (SR05, Female, 36, Bangladeshi).

While the pandemic meant that opportunities for holidays and social gatherings were missed by many, the cancellation of international travel was particularly difficult for those with close family living abroad. The circumstances of the pandemic in other national contexts could also add stress:

All of my mum’s side are in India and […] initially it was full lockdown and then there was news of police brutality and then there was news of how bad [high] the numbers [of cases] were, people hijacking food trucks and all this. And obviously I’m just thinking, ‘Oh my god! My family. What is it like for them?’ (IP02, Female, 28, British Indian)

People’s lockdown experiences were also affected by a fear of negative treatment. Some people described personal experiences of racially-motivated poor treatment, in this case in line with other research identifying biased policing during the pandemic (Busby and Gidda, 2020, Harris et al., 2021a; b):

I was stopped 3 times during lockdown by police and PCSO [Police Community Support Officer] while out with my 3 year old daughter, none of my white mum friends were (SR38, Female, 47, Indian).

But others described how their fear of racist victimisation had affected their lives and their mental health. Some participants reacted to these fears by adopting vigilant coping behaviours, such as reducing their social contact.

I have some friends who are of East Asian decent and they’ve experienced racial attacks—being made to feel like they were the cause for the pandemic. […] even though I’m African, I initially felt safer to stay home and on edge of [because I was worried about] what effect the pandemic would have on racial tensions (SR03, Female, 23, Black African).

While acknowledging that some people experienced an additional risk to which she was not exposed, SR03 also identifies a wider racism endemic to British society which has been exacerbated by the pandemic, and of which all those in racialised groups are at risk. People’s fear of exposure to racist violence also increased with lockdown restrictions on movement, a loss of witnesses potentially making violence more likely as well as reducing opportunities to respond effectively if it did.

Hate can be quite frightening, and […] your children are walking the street at night, knowing there could be anyone like that walking on the streets. It did get quite frightening because of the isolation of the lockdown, not so many witnesses if you’re on the street for any aggression or violence (IP07, Female, 53, Black Caribbean).

Experiences of racism in one domain could also produce fears about negative treatment in others. In the quotation below, and as with SR03 above, the articulation of racism by members of the public produced concerns regarding the ubiquitous nature of racism in Britain, including its presence among those providing healthcare. This heightened people’s sense of stress and also their perceived need for extra vigilance in behaviours to reduce the risk of infection:

With BLM […] I found a lot of bigots, and a lot of negativity, a lot of racism [online…] which didn’t make me feel confident that if I went out and asked for support, or if my health failed and I needed support and I came across someone that was really against BLM and was very angry because they’d had a protest that they were going to treat me [kindly]–so I kept myself as safe as possible because there was no way I was entering into a hospital. […] it played on your mental health a lot. (IP07, Female, 53, Black Caribbean)

Racism could directly undermine people’s ability to develop supportive networks with their neighbours, which limited people’s sense of available support during the lockdown:

[The] white middle-class neighbourhood helped each other. They didn’t help me […] I thought, ‘Even at the lowest point, where we could all die […] they still have that racism.’ (IP07, Female, 53, Black Caribbean)

In the absence of support from her local neighbourhood, IP07 established a “community” of people with similar ethnicities living in other areas. This network could not only provide the practical and emotional support she needed during the pandemic but also offered connections stemming from common experiences of social exclusion:

My community is extended—Black professionals and the Black communities that I live close to, that I go to for food, warmth, emotional warmth […] And those are the people that kept my sanity, and those are the people that understand that we are at the bottom of the chain (IP07, Female, 53, Black Caribbean)

A fear of racism could also impact on someone’s access to social support. IP04 describes how such anxieties had prevented them from even trying to initiate social connections:

…because English is not your first language, your name isn’t familiar, you probably don’t talk in the same accent as people do here, so it’s really difficult to adjust yourself to that kind of life. At times, when you feel those negative things [attitudes], you feel like “oh probably everybody is the same’ but then when positive things happen, you start thinking ‘oh no, there are still good and nice people around”. […] When somebody does a nice gesture for you, you feel overwhelmed. (IP04, Female, 42, Kashmiri)

As before, negative experiences identified in one domain increased a perceived need for vigilance in others, which led them to avoid making efforts to engage with people with whom they were unfamiliar despite the potential benefits of such relationships. While this participant does not explicitly mention racism, the “negative things” she has experienced relate to characteristics which would mark her as culturally different. Moreover, her sense of being “overwhelmed” by any “nice gestures” suggests that her sense of vulnerability to racism is significant. As discussed earlier, this may have occurred as a consequence of previous personal experiences of racist violence or through the development of a perception based on the experiences of others—or wider public discourses—that such reactions are commonly experienced by people with similar characteristics to hers.

### The Impact of a Heightened Sense of Covid-19 Risk

People’s pandemic anxiety was exacerbated for those, or with family, with health conditions considered to increase the risk of Covid-19 complications, particularly when they worked in a role which they could not provide at home. This concern increased with news regarding the higher prevalence of Covid-19 infections and deaths amongst those in minoritized ethnic groups.

It has been a constant worry with my husband and eldest son going to work. It has been a huge worry knowing that BAME people are at a higher risk. It has just been a huge worry overall and it has battered my mental health (SR41, Female, 36, Mixed—Black Caribbean and white).

Awareness of this additional health risk encouraged people to go to more extreme measures to protect themselves, even if this could exacerbate their social isolation.

I wasn’t worried too much before and at the start of lockdown. However since the news of BAME people being much more likely to be impacted I have been very worried—I rarely leave the house and it has caused me anxiety for the first time in my life (SR39, Male, 37, Mixed—Black African and white).

People felt that the Government’s pandemic planning did not acknowledge, and might actually increase, these risks:

I have had some increased anxieties since lockdown rules have relaxed and am aware of higher risks for BAME people (SR24, Female, 42, Indian).

Some of the participants unable to work from home during the pandemic felt empowered to negotiate with their employers to ensure their safety at work, should this become necessary. But there was a concern that not everyone had that same capacity, or “voice”.

Nobody would force me to go into an office or do anything I didn’t want to do […] But like I said I’m probably in a better position than most because I’ve got a voice. So if you make me do something I’m not doing it and I can give you reasons why—and I’ll use [my knowledge of] policy to protect myself. I think if I was more of a vulnerable person and I had to work such as a taxi driver, or a person who was working frontline in a shop and I had to work or I wouldn’t get paid, it would be a different position (IP07, Female, 53, Black Caribbean).

Here, and in the quotation below, there is an awareness of the consequences of the occupational concentration which is argued to help explain ethnic inequalities in Covid-19 infections and deaths (Platt and Warwick, 2020a,b, BSWN, 2020):

As an African migrant, a lot of people tend to work as healthcare assistants, nurses, doctors, so just knowing that a lot of my family members were out there on the frontline, there was anxiety on that front and hoping that they remain safe. I think the knowledge as well that there’s higher prevalence in BAME communities—obviously that kind of added to the anxiety (IP01, Female, 25, Black African).

These comments may suggest that all those working in such roles may be exposed to a similar risk—regardless of ethnicity. However, the comments of some survey respondents suggested a particular lack of empowerment among those in minoritized ethnic groups in some occupations which may help explain ethnic inequalities in infections and deaths even within particular occupations (Cook et al., 2020). These people argued that they had been placed at unnecessary risk by their employer in a way which was not experienced by white colleagues.

I am the only BAME employee in my service. […] I had to raise a complaint as I felt I was being put at risk not in line with that expected of my white colleagues despite [me] having increased risk [established through a workplace risk assessment] (SR17, Female, 30, Mixed—Asian and white).

I was given more responsibility to lighten the load [on] other senior members of staff […] I was offered no flexibility […] WFH [working from home] was only for the senior staff, whom are white (SR21, Female, 43, Indian).

In addition to problems with the actions of particular employers, participants also described Government failures to provide to support to those at additional risk: that “Black people who are on the frontline have the least protection and are not given any dedicated support.” (SR33, Female, 36, Black African). While the Government had introduced new measures designed to assess the risk posed to those in minoritized ethnic groups in the workplace, these measures placed additional burden on staff and could directly increase people’s sense of vulnerability to infection. Moreover, some people felt that such measures were simply “tick box” exercises, which had no practical value and only served the Government by giving people the impression that were taking appropriate action when they were not:

I’ve had extra risk assessments at work. […] it just became a big deal [inconvenience] anytime I did a home visit and then because I was having these extra risk assessments, it made me more nervous to do these home visits. […] I feel like I’m just in a box and being asked for a tick-box exercise rather than anything else. (IP02, Female, 28, British Indian)

This distrust in the Government’s motivations was also raised in relation to other aspects of the pandemic, as we shall discuss in the next section.

### The Causes and Consequences of Inadequate Government Responses to the Pandemic

Every participant expressed shock and deep frustration at the Government’s handling of the pandemic, which had significantly undermined their “trust”. Having family and friends living in other national contexts offered participants a particularly clear vantage point from which to recognise specific opportunities the Government had missed to act quickly and effectively in the face of rising infections and deaths, rather than waiting till “after the horse had bolted”. The Government’s inaction was particularly frustrating to those whose work enabled them to see first-hand the dire need for rapid action.

Don’t get me started. The Government—working in the field I’m in, I saw cases […] and we had to sit and wait for the Government to tell us what to do—by then it’s too late (IP09, Female, 56, Black Caribbean).

Government and media discussions regarding the causes of the pandemic—which ignored the role played by long-term Government policy in the escalation of the problem—reinforced this sense of dishonesty and corruption. Participants believed that members of the scientific community had been bribed to distort the truth, “paid back-handed to tell me rubbish”. Again, these issues were particularly frustrating to those that worked in health or social care, or had friends and relatives in nursing homes or hospitals.

In a nursing home […] that’s where we saw the lack of PPE […] The media going out there [“blah blah”], we just thought “lies, lies, lies”, all the way through. Then you go through anger [get angry] because obviously they then blame nursing and care staff and you knew that nursing and care staff did an incredible job.[…] So it was just the “blame game” […] You [Government] knew it [what was happening] but […] you were too busy covering your own asses (IP07, Female, 53, Black Caribbean).

Participants described media reports of ethnic inequalities in Covid-19 risk as particularly unhelpful and “sensationalist”. As a consequence, they felt they had received little valuable information regarding the reasons for these differences, how to protect themselves or the Government’s “action plan” to respond to them. This “insufficient guidance/research and action” was felt to expose “the Government’s lack of priority [to protect people in ethnic minority groups] even though we appear to be the worst affected” (SR59, Female, 37, Black African).

Explanations offered for ethnic inequalities in Covid-19 infections and deaths, when they occurred, were described as “disgustingly divisive”. There was a concern that these had focused on genetic/biological factors and ignored the structural factors which actually explained these greater risks. For some participants, it was clear that the issue “was less about race and more about postcodes” (SR56, Female, 34, Pakistani) and other societal factors. They took this apparent distortion of the evidence as further indication of the Government’s dishonesty.

[The message that] ‘you [those in ethnic minority groups] basically have [problems with] your metabolism or your body make-up and it’s totally different [to other people’s] so you’re going to be high profile [at high risk] for Covid-19′ was bull[shit]—we just thought “you weren’t treating us properly”. […] You know it’s not that [explanation. Actually,] it’s lack of resources, PPE, and maybe lack of trust, lack of good quality of health[care], so there were more reasons behind it [than they were admitting] (IP07, Female, 53, Black Caribbean).

Others highlighted the shortcomings in this framing by simply asking, “Since when was a virus racist?” (SR49, Female, 34, Mixed—Black Caribbean and white). Even where these reports included greater acknowledgement of the significance of structural factors, they did not always offer sufficient analytical depth to allay people’s fears or confusion:

It was almost like sensationalist reporting in that ‘you’re more likely to die of Covid or contract covid if you’re BAME′ and they didn’t say why. One local reporter said, it’s because these groups don’t often have access to a garden. And I’m like, ‘well, I’ve always had a garden. So it was all this sort of rubbish, because the likely explanation doesn’t put the Government in a good light […] If there’s no why or understanding then you’re scaremongering. You’re not really helping the situation. […] I’m isolating by myself in a three-bed semi-detached [house] with a garden. I can’t be the only [Black] person [like that]. So it would be interesting to see, is it BAME living in poorer conditions, you know high rise [accommodation]? What is it? (IP05, Female, 39, Black British Caribbean)

Even though this participant recognises her more privileged socioeconomic position, the media report did not support them to fully appreciate whether and how this might enable a reduced vulnerability to Covid-19 infection, or its more extreme consequences. Again, there is a perception that the Government and media would purposefully withhold information to avoid exposing their contribution to the problem. IP05 also reflected on the tendency for the Government and media to present a very homogenised, negative and biased portrayal of those in minoritized ethnic groups:

I’m Black but I don’t live in the poorest suburb of (area). I drive, I’m in this [higher] pay bracket, where am I? Unless I’m Black, single, single mother, on benefits, you know all the kind of negatives—I don’t exist. And I go, ‘well, I know I’m not unusual and there are others, so where are we (IP05, Female, 39, Black British Caribbean).

This homogenising discourse further reinforced a sense that ethnic inequalities in Covid-19 were driven by genetic/biological risks, and immune to the effects of socioeconomic and other privilege. It was also felt unhelpful for people trying to determine their own level of pandemic risk:

I find that a lot of the discussion around “BAME” [Black, Asian and Minority Ethnic] during this pandemic relates to Black or Black&Asian [people], but doesn’t consider other groups. […] I feel I always have to check if I’m included or not. (SR22, Female, 45, Mixed—other)

As a mixed-race person I have struggled at times to know whether and to what extent advice related to increased risk for “BAME” people applies to me (SR17, Female, 30, Mixed—Asian and white).

More generally, people described how their own pandemic response had been undermined by confusing and contradictory Government messaging which forced people to make their own decisions about how to protect themselves. This distrust of the Government’s motives also encouraged a perceived need for additional vigilance, to “use your [own] intelligence, not just listen to what the Government is telling you”. People described seeking alternative and, what were perceived to be, more reliable sources of information on how to manage their risk than the Government was providing.

We created a bubble long before the Government told us we could because we knew we were going to have to protect ourselves. There was no way, as a Black person, I was going to take on anything that white upper-class person was going to tell me, because they weren’t going to have any thoughts on my behaviour or what was going to help me (IP07, Female, 53, Black Caribbean).

This distrust of the Government’s motivations could also affect people’s engagement with other aspects of the Government’s response to the pandemic, for example in relation to the Covid-19 vaccine, voicing suspicions and concerns which have also been raised by NHS staff (Woodhead et al., 2021):

Now we’re back to “[…] we want Black people to come in first and have the injections because you’re at highest risk”. How many idiots do you think are out there that are going to be Black–including myself–that are going to have an injection by our glorious leader [Boris Johnson] that made so many mistakes at the beginning? And then we have a glorious leader [Donald Trump] in America giving out the same message–“you’re nothing, you’re rubbish, I think nothing of you, but you’re going to have the injection first and then we’re going into Africa”. (IP07, Female, 53, Black Caribbean)

While it is unclear whether this concern led to a refusal to receive the vaccine, it is likely to have contributed to a greater sense of hesitancy, and additional stress amongst those receiving the vaccine. There is a strong sense here of the ways in which racism perceived in different contexts, even when experienced indirectly (eg through the comments and actions of Donald Trump), can combine to introduce/perpetuate a personal sense of vulnerability, which may even directly exacerbate a health risk, through disengagement with effective pandemic protection strategies.

Approaches which failed to acknowledge the diversity within and between minoritized ethnic groups encouraged a sense that they were inherently different from the rest of (white) British society. Participants argued that this enabled them to be presented (by the Government and media) as a threat to (the health of) British society, which could directly motivate racist violence:

I don’t like being classed as “BAME” […] since Covid it has underlined [reinforced] the racism [existing in Britain] where people are […] making it known [encouraging people to think] that we are carriers. “BAME” I see as classed as ‘others’. (SR19, Female, 56, Black Caribbean)

People felt that the pandemic had been used to try to galvanise the white British public, through the further marginalisation of minoritized ethnic groups and to serve an anti-immigrant agenda:

Worryingly is the narrative that shifted from “white people pulling together and fighting this through”, to members of the BAME community targeted as the major carriers and causing harm and being reckless. It was ironic that once the PM overcame the virus, his first point of business was tackling immigration, never mind the fact that many non-whites died looking after their elderly. [It] speaks volumes (SR44, Male, 50, Black Caribbean).

As such, rather than ensuring the protection of the whole population, the Government had “turn[ed the pandemic] into a race war” (SR49, Female, 34, Mixed—Black Caribbean and white).

Other events occurring during this time were also felt to have been misappropriated to reinforce this sense that those in minoritized ethnic groups are inherently problematic and dangerous. The Government and media were argued to have purposefully misrepresented the Black Lives Matter movement protests occurring in response to the death of George Floyd in order to “politicise” them and reinforce a sense that those in minoritized ethnic groups were to blame for their own difficulties, in part by minimising the attention given to the risky activities of people in other ethnic groups.

I think there’s a bit of a blame sometimes—so like the Black Lives Matter protest. The news really focused on how ‘this is really going to help the surge of coronavirus, the numbers are going to go up and up and up’ and actually everyone on that protest was keeping their distance. They were wearing masks. As much as they could do at protests, they were following rules. And then you’ve got those pictures of Brighton where you’ve got hundreds and hundreds of people hoarding on the beach and then nothing [was said] (IP02, Female, 28, British Indian).

People were also angry that this victim-blaming had enabled the Government and media to impede their ability to express their legitimate concerns about the scale of racism in Britain: “It felt like the pandemic was being used to stop people from going out to protest.” (IP01, Female, 25, Black African).

Like Elias et al. (2021), people did not see these negative depictions as incidents isolated from the negative treatment of people in minoritized ethnic groups in other contexts and times. People described how negative representations in relation to the pandemic replicated and were enabled by earlier discourses about those in racialised groups.

With Brexit, the racism has risen and people have been attacked […] I’ve grown up with all the racism and to keep seeing it and hearing it—it just seems like it’s never going to go (IP09, Female, 56, Black Caribbean).

We stem from hate and we’re still moving through hate. [… like] Nigel Farage who stokes things so badly and has these sheep behind him. And then you have the people who take it further, is it the Christchurch murderer who killed 50-odd people in a mosque (IP07, Female, 53, Black Caribbean).

These quotations described the legacies of racism experienced since childhood, which influence and are reproduced in later racist tropes, for example, during Brexit. They also reflect on the significant role of commentators like anti-immigrant United Kingdom Independence Party (UKIP) leader Nigel Farage and other political leaders in encouraging the racist violence perpetrated by others—the “sheep” who follow them unquestioningly, sometimes to perform acts of massacre such as the deaths of 50 people following shootings at two mosques in Christchurch, New Zealand, on March 15, 2019. It was argued that this political atmosphere presented opportunities which were then exploited by the Government during the pandemic.

So they go through [they thought] “let’s just blame them [Black people] because it’s easier” because at the moment, we’re in the right-wing place. So let’s just keep it there because people will believe that (IP07, Female, 53, Black Caribbean).

The increase in (awareness of) incidents of racism during the pandemic had a significant impact on people’s wellbeing. People explained “the impact of systematic racism in the world, this has affected me a lot” (SR42, Female, 56, Black—other) and that the increase in “race related attacks has been hard to watch and endure” (SR33, Female, 36, Black African). Even when occurring in contexts distant from their own, this awareness could encourage people to reflect on themselves as members of a racialised group, and the inescapable nature of and risks associated with this. Direct and indirectly experienced incidents of racism occurring during the pandemic could become “triggers” for earlier negative experiences which exacerbated people’s mental distress.

The triggers from the media, BLM [Black Lives Matter protests] and BLM happening at the same time as Covid–[…] through lockdown, I’ve had time to reflect and through BLM I’ve seen a lot more of the racism that we face […] You face it daily […] Everything you do you have to think about your skin colour because you know you may be treated differently (IP07, Female, 53, Black Caribbean).

Similarly, IP01 describes the way in which reflecting on previous experiences of negative treatment, motivated by experiences in relation to the death of George Floyd and the Black Lives Matter protests and ethnic inequalities in Covid-19, had produced “a lot of anger in the community” as it has exposed the persistence of these issues as a consequence of a lack of meaningful action to address them among those with the power to do so:

For a lot of people it [BLM] brought up past experiences of racism which people had brushed under the carpet. I think it brought to the forefront some of the issues of racism that is experienced by Black people in particular. So, I think a related issue was obviously the higher prevalence of coronavirus deaths within the Black community. I think it added to the rhetoric of the BLM movement’ (IP01, Female, 25, Black African).

The pandemic, then, as well as a specific event, became the latest manifestation of the negative treatment which those in minoritized ethnic groups had to continuously negotiate, as “second class citizen[s]”. This final quote is a testament to the perpetual effort involved in maintaining resilience in the face of these constant threats of victimisation, which come from all sectors of society:

They were trying to make you a victim and they didn’t succeed because you were always one step ahead of them, like you [always] are, because you have to be, in work or in life. You’re always one step ahead of a racist. It was just amazing the path they took. And with straight faces (IP07, Female, 53, Black Caribbean).

Despite this long history, this participant still expresses disbelief that, during the Covid-19 pandemic, the British Government could behave so abominably so brazenly, ie “with straight faces”, without any concern for negative repercussions. Not surprisingly, this offered them little hope for things improving in the future.

## Conclusion

These findings offer a valuable insight into the experiences of people in different minoritized ethnic groups during the first Covid-19 lockdown in South-West England in 2020. Initially, some participants, shielded from the more severe financial consequences of the pandemic and its lockdown, described positive experiences including opportunities for personal growth. In itself, this may not be considered a particularly novel finding. However, it takes on new significance in light of popular discourses, referenced by our participants, which present those in racialised groups as inherently different from wider British society. Yet, the influence of “ethnicity” still looms large. A sense of greater health risk—to yourself, your friends and family and other people “like you”–developed as evidence emerged of persistent ethnic inequalities in Covid-19 infections and deaths. These concerns were compounded by knowledge of the concentration of those in minoritized ethnic groups in exposed occupations or with particular co-morbidities. But there is also evidence of a particular sense of threat which is not rooted in the pandemic itself. That while the virus may not be “racist”, the implications for people’s pandemic experience of living in a society where racist victimisation is considered endemic are expressed very clearly. On top of a general sense of Government incompetence, corruption and distrust, people described a racism which infected policy and behaviour at a national level. A racist media which spun stories which deliberately delegitimised and demonised peaceful and justified protests and the ignored the actual drivers of higher infections and deaths to blame the victims. Statutory authorities who failed to protect. And a public—justified by behaviour from the top—who used the pandemic and protests as opportunities for racist violence.

The consequences of direct experience of poor treatment are easily understood. But these findings also effectively illustrate the nature of lives lived with the threat of violence, even if that threat is not always actually realised. A fear of going out in public for fear of being attacked. A fear of needing to rely on people for protection, information or care who would deliberately act to harm you, whether that be neighbours, the police, healthcare providers or the Government. Concerns which led to greater mental strain both directly and indirectly, including as a consequence of the various forms of vigilance people adopted in response. This research offers further evidence of the need to recognise the inter-connected and reinforcing nature of incidents of racism. The pandemic and other racisms experienced—vicariously or personally—at this time (including the death of George Floyd and the actions it prompted) combined to paint a stark picture of the risk experienced by all those in racialised groups, in Britain and elsewhere. These incidents also became “triggers” which forced people to reflect on their own histories as members of these groups. The pandemic, then, became not a snapshot of a time like no-other, but testament to the past and an insight into the risks of the future.

While this paper offers valuable insights into the pandemic experiences of those in minoritized ethnic groups, the sample is small and select. As such, these findings are not generalisable to a wider population and we cannot establish the extent to which these experiences may be common. In particular, the impact of vulnerability to poverty on experiences of lockdown remains a significant part of the pandemic experience of many, disproportionately those in minoritized ethnic groups. Interview participants in this study were relatively privileged in terms of career, socioeconomic position, English-language ability, citizenship/migration status, and digital connectivity. This limits the potential for this study to offer insights into this aspect of pandemic experience and this must be the focus of further research. There is also a need for further research which can explore more directly the ways in which this pandemic experience is gendered (Laster-Pirtle and Wright, 2021). That said, it may be argued that this research offers a valuable opportunity to not focus on, what one participant called, “all the negatives”, approaches which may in themselves encourage the sense that those in minoritized ethnic groups are inherently different and problematic, compared with the white British majority. Moreover, these findings show that while economic security enables some potential commonalities in pandemic experience with wider British society, it does not protect people from all the health risks to which those in minoritized ethnic groups are exposed. Our findings concur with existing literature regarding the range of ways in which racism can impact on people’s lives and wellbeing.

Despite centuries spent trying to find it, there remains no solid evidence that ethnic inequalities in the vast majority of health or other social and economic circumstances can be explained by physical or cultural differences between the groups. All other things being equal, the pandemic experiences of the British public *should* be complimentary. Our participants see this clearly. Railing against persistent victim-blaming discourses, they instead draw attention to the role of structural/societal factors: racism; a lack of economic resources, social support and effective healthcare; a reduced ability to protect yourself in the face of vulnerabilities; and people’s reactions to the lack of trust these engender. If we are to have a hope of successfully building a fairer, healthier, inclusive and sustainable society in the aftermath of the pandemic, we must, as a minimum, insist that political, media and academic discourses are similarly reflective regarding the causes of these inequalities.

We must also acknowledge the centrality of racism to the experiences of many people in minoritized ethnic groups living in the United Kingdom, and elsewhere. It is a key driver of the inequalities in economic and other experience so significant for ethnic inequalities in Covid-19 infections and deaths (PHE, 2020a; Becares and Nazroo, 2020; Godlee, 2020). This research suggests that racism has also played more a direct and prominent role in the pandemic experiences of people in minoritized ethnic groups. While many of us experienced fear as Covid-19 spread around the globe, this fear was exacerbated among those who not only felt they did not have the attention of those with the power to protect them, but that they might actually sacrifice them to save themselves. Sewell et al. (CRED, 2021) are correct to argue that people’s sense of racism may be forged in history. But this research suggests that this history continues to repeat itself. For many, the pandemic has served as further evidence of the need for those in ethnic minority groups to maintain a healthy sense of distrust to survive, and be mindful that “the racist” remains close behind.

## Data Availability

The data presented in this article are not readily available because this would contravene the terms of the ethical agreements under which the research was conducted. Requests to access the data should be directed to SK, saffron.karlsen@bristol.ac.uk.

## Footnotes

1 Lockdown is the cessation of all non-essential activities to slow the spread of Covid-19. In the United Kingdom, this saw strict limits imposed on people’s activities both within and outside of their homes, including both what they could do and who they could spend time with. The first United Kingdom lockdown was applied on 23 March 2020, lasting until 4 July 2020, although regional/territory specific approaches were introduced after this time. Subsequent national lockdowns were implemented in October 2020 and January 2021, although with differing degrees of impact (e.g., regarding whether or not educational establishments were affected) and territorial timeframes.
